# Supplementary material for: The impact of organizational culture on professional fulfillment and burnout in an academic department of medicine
Source: PLoS One. 2021 Jun 9;16(6):e0252778. doi: 10.1371/journal.pone.0252778 (PMC8189486; doi:10.1371/journal.pone.0252778)
Supplement: S1 File — (PDF) [file pone.0252778.s001.pdf]

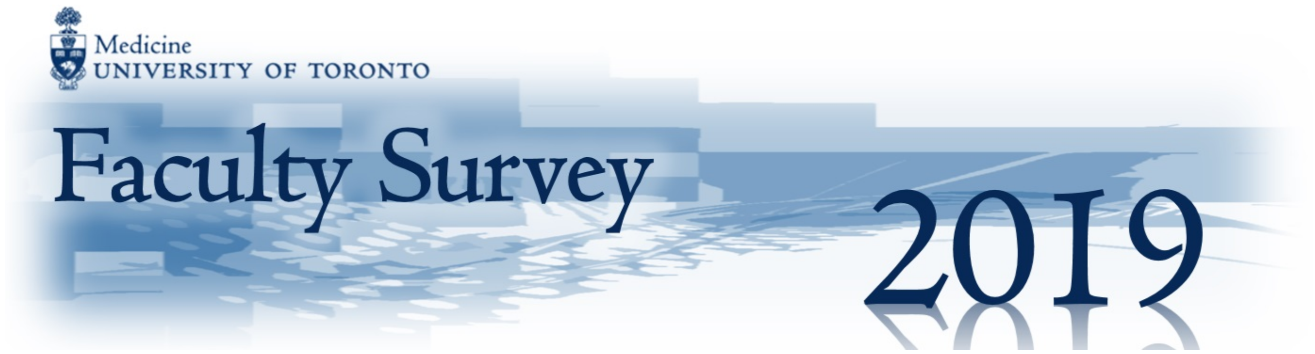

Department of Medicine (DoM) Faculty Survey 2019

CONSENT TO PARTICIPATE

You may start and return to this survey over multiple sittings, at your convenience. Your answers on a completed page are preserved, and when you next click on the survey link, you will be taken to the next section following your last completed page. You can change your answers on any survey page(s) until you click 'submit' at the end.

*** 1. Confirmation**

○ I wish to continue and complete the survey to help inform the Department of Medicine (DoM) on issues related to Career/Work Place Satisfaction.

○ I wish to opt-out of this survey.

Medicine
UNIVERSITY OF TORONTO

Faculty Survey 2019

Department of Medicine (DoM) Faculty Survey 2019

INSTRUCTIONS & NOTES

We anticipate that this iteration of the Faculty Survey will take approximately 15 minutes to complete.

When completing this survey, if you are cross-appointed to two or more divisions, we ask that you answer questions from the perspective of the SINGLE division with which you most identify (i.e. the division in which you spend more clinical time, have your most meaningful scholarly activity, and conduct most of your mentoring).

Abbreviations / Clarification of terms:

- PIC: Physician-in-Chief, the head of the hospital department of medicine
- DoM: Department of Medicine
- DDD: Departmental Division Director (Citywide), the head of the citywide division to which you belong
- Hospital Division Head (Local), the head of the hospital division to which you belong
- CIHR: Canadian Institutes of Health Research
- CFA: Clinical Faculty Advocate (See: http://www.deptmedicine.utoronto.ca/clinical-faculty-advocate)

Medicine
UNIVERSITY OF TORONTO

Faculty Survey 2019

Department of Medicine (DoM) Faculty Survey 2019

SECTION 1: WORK-LIFE INTEGRATION & WELLNESS

[Definition of Work-Life Integration.]

The following questions seek to assess your career satisfaction as a member of the DoM and is based on the Stanford Professional Fulfillment Index which is a validated wellness screening tool (Trockel et al., 2017).

2. How true do you think the following statements are about you at work during the past two weeks?
Select ONE response for each option provided.

	Not at all True	Somewhat True	Moderately True	Very True	Completely True
I feel happy at work	○	○	○	○	○
I feel worthwhile at work	○	○	○	○	○
My work is satisfying to me	○	○	○	○	○
I feel in control when dealing with difficult problems at work	○	○	○	○	○
My work is meaningful to me	○	○	○	○	○
I'm contributing professionally (eg. Patient care, teaching, research, and leadership) in the ways I value most	○	○	○	○	○

3. During the past two representative (clinical / scholarly) weeks I have felt…
Select ONE response for each option provided.

	Not at all	Very little	Moderately	A Lot	Extremely
A sense of dread when I think about work I have to do	○	○	○	○	○
Physically exhausted at work	○	○	○	○	○
Lacking in enthusiasm at work	○	○	○	○	○
Emotionally exhausted at work	○	○	○	○	○

4. During the past two representative (clinical / scholarly) weeks, my job has contributed to me feeling…

Select ONE response for each option provided.

	Not at all	Very little	Moderately	A Lot	Extremely
Less empathetic with my patients	○	○	○	○	○
Less empathetic with my colleagues	○	○	○	○	○
Less sensitive to others' feelings / emotions	○	○	○	○	○
Less interested in talking with my patients	○	○	○	○	○
Less connected with my patients	○	○	○	○	○
Less connected with my colleagues	○	○	○	○	○

Medicine
UNIVERSITY OF TORONTO

Faculty Survey 2019

Department of Medicine (DoM) Faculty Survey 2019

SECTION 2: CIVILITY / PROFESSIONALISM

The following questions address issues related to equity, diversity and professionalism.
Click here for Glossary reference.

5. Please rate your satisfaction with the efforts made by… to create a collegial and supportive environment that promotes inclusivity.
Select ONE response for each option provided. Note: This question is based on your satisfaction with the efforts made, and not your overall satisfaction with the environment itself.

	Not Applicable	Strongly Dissatisfied	Somewhat Dissatisfied	Neutral	Somewhat Satisfied	Strongly Satisfied
University DoM	◯	◯	◯	◯	◯	◯
University Division	◯	◯	◯	◯	◯	◯
Your Primary Hospital DoM	◯	◯	◯	◯	◯	◯
Your Primary Hospital Division	◯	◯	◯	◯	◯	◯

6. The people I work with at (my) … interact with me in a respectful and civil manner.
Select ONE response for each option provided.

	Not Applicable	Strongly Disagree	Somewhat Disagree	Neutral	Somewhat Agree	Strongly Agree
University DoM	◯	◯	◯	◯	◯	◯
University Division	◯	◯	◯	◯	◯	◯
My Primary Hospital DoM	◯	◯	◯	◯	◯	◯
My Primary Hospital Division	◯	◯	◯	◯	◯	◯

7. I am aware of policies and procedures in place at … to address unprofessionalism (for example, issues of disrespect, abuse, bullying, microaggression, and discrimination).
Select ONE response for each option provided.

	No	Uncertain	Yes	N/A (Not Applicable)
University DoM	◯	◯	◯	◯
My Primary Hospital	◯	◯	◯	◯

8. Please rate your confidence that you can take action to address unprofessionalism at … without concern for reprisal.
Select ONE response for each option provided.

	Not Applicable	Very Doubtful	Somewhat Doubtful	Neither doubtful nor confident	Somewhat Confident	Strongly Confident
University DoM	◯	◯	◯	◯	◯	◯
University Division	◯	◯	◯	◯	◯	◯
My Primary Hospital DoM	◯	◯	◯	◯	◯	◯
My Primary Hospital Division	◯	◯	◯	◯	◯	◯

*** 9. Have you witnessed what you perceive to be “unprofessionalism” (for example, issues of disrespect, abuse, bullying, microaggression, and discrimination) by faculty members, towards others (for example trainees, other academic faculty within the department or otherwise, interprofessional colleagues) at your hospital or at the university, within the last two years?**
Select ONE response only.

◯ Yes

◯ No

*** 10. Have you personally experienced what you perceive to be "unprofessionalism" (for example, issues of disrespect, abuse, bullying, microaggression, and discrimination) in the Department (at your hospital or at the university) within the last two years?**
Select ONE response only.

◯ Yes

◯ No

11. Please feel free to provide any comments to elaborate on your answers to any of the questions on this survey page.

Medicine
UNIVERSITY OF TORONTO

Faculty Survey 2019

Department of Medicine (DoM) Faculty Survey 2019

SECTION 2: CIVILITY / PROFESSIONALISM (PERSONAL EXPERIENCE)

The following questions refer to your collective experiences with unprofessionalism.

12. What types or form(s) of unprofessionalism did these events (collectively) include?
Select ALL responses that apply.

- [ ] Disrespect
- [ ] Abuse
- [ ] Bullying
- [ ] Microaggression
- [ ] Discrimination
- [ ] Other (please specify)

13. Did you perceive any of these events to be attributed to your identification with a specific minority group?
Select ALL responses that apply.

- [ ] No
- [ ] Gender
- [ ] Race/Ethnicity
- [ ] Sexual Orientation
- [ ] Other (please specify)

14. What was the impact of these events on you personally or professionally? Please comment.
If you want to access resources for support or additional guidance, please click here.

*** 15. Did you take action/report any of the events to a direct supervisor at the time?**
Select ONE response only.

- ( ) Yes
- ( ) No

16. Comments in regards to taking action/reporting:

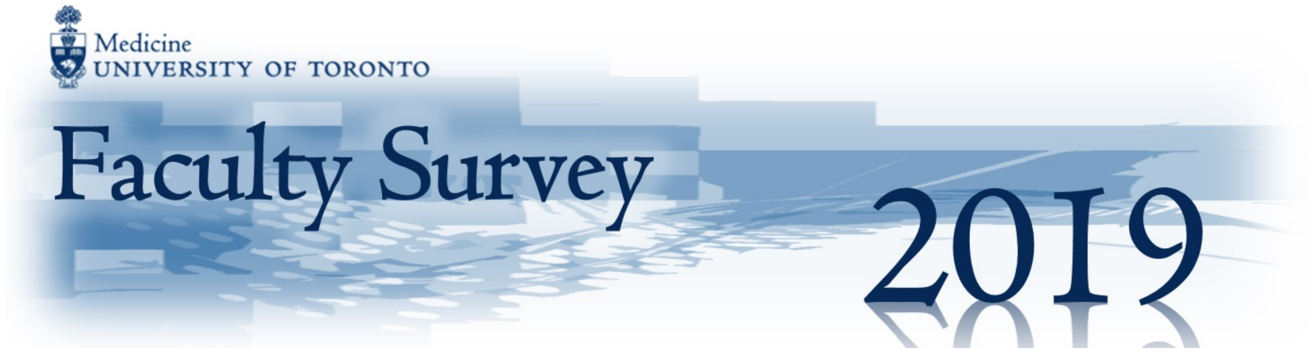

Department of Medicine (DoM) Faculty Survey 2019

SECTION 2: CIVILITY / PROFESSIONALISM (REPORTING)

17. Did you experience any reprisal after you reported any of the events?
Select ONE response only.

- ◯ Yes
- ◯ No

Comments

[ ]

18. To your knowledge, was there any action taken as a result of your complaint (e.g., investigation)?
Select ONE response only.

- ◯ I don't know
- ◯ Yes
- ◯ No

Medicine
UNIVERSITY OF TORONTO

Faculty Survey 2019

Department of Medicine (DoM) Faculty Survey 2019

SECTION 2: CIVILITY / PROFESSIONALISM (Cont'd)

19. Have you had any formal unconscious bias training (e.g., Harvard Implicit Association Test, or other similar training) within the last two years?
Select ONE response only.

- ◯ Yes
- ◯ No

Medicine
UNIVERSITY OF TORONTO

Faculty Survey 2019

Department of Medicine (DoM) Faculty Survey 2019

SECTION 3: WORK STRUCTURE

The following questions seek to understand how work structures, including schedules, remuneration and benefits, leaves of absence, meetings, and electronic tools, affect the experiences and wellness of faculty members.

20. Are you interested in reducing your clinical workload to help with yourwork-life integration?
Select ONE response only.

- ○ Yes
- ○ No
- ○ Not sure

SECTION 3: WORK STRUCTURE (Cont'd)

21. Please rate your level of interest in the following strategies to enable you to reduce your clinical workload.
Please select ONE response for each option provided.

	Definitely not Interested	Slightly Interested	Moderately Interested	Very Interested	Extremely Interested
Shift Work (e.g., defined hours of work over days / evenings / weekends / nights)	◯	◯	◯	◯	◯
Part-time work	◯	◯	◯	◯	◯
Work Sharing (2 individuals sharing a full-time position)	◯	◯	◯	◯	◯
Reducing clinical hours by delegating part of the clinical workload to advanced practice providers (e.g., Nurse practitioners or Physician assistants)	◯	◯	◯	◯	◯
Duty hour restrictions (e.g. having different staff provide day versus night coverage)	◯	◯	◯	◯	◯
Alternative funding models to reduce dependence on fee for service income (e.g. mixed stipend + fee for service)	◯	◯	◯	◯	◯
Expanded faculty numbers	◯	◯	◯	◯	◯

Comments

[ ]

22. Please indicate your willingness to accept the following compromises that may need to be undertaken to help with your work-life integration.

Select ONE response for each option provided.

	Willing to accept	Not willing to accept
Reduction in total income	○	○
Reduction in clinical income	○	○
Reduction in clinical hours	○	○
Becoming a salaried employee	○	○
Reduction in continuity (with patients)	○	○
Reduction in continuity (with trainees)	○	○
Delayed CFAR [4] or promotion	○	○

Comments

[4] *CFAR = continuing faculty appointment review (3-year review).*

Medicine
UNIVERSITY OF TORONTO

Faculty Survey 2019

Department of Medicine (DoM) Faculty Survey 2019

SECTION 3: WORK STRUCTURE (Cont'd)

23. Would you be interested in being remunerated with a "fixed salary" (akin to working as an employee)?
Select ONE response only.

- ◯ Yes
- ◯ No
- ◯ Not sure
- ◯ It depends, please provide a comment

24. Are you aware of policies and procedures within your practice plan to undertake a parental leave?
Select ONE response only.

- ◯ Yes
- ◯ No

Comments

25. Please indicate if you think the following types of leave should be formally accommodated by your hospital DoM (which may require contributing more towards the practice plan)?
Select ONE response for each option provided.

	Yes	No
Parental leave	◯	◯
Elder-care leave	◯	◯
Child-care leave	◯	◯
Personal emergency leave	◯	◯
Family emergency leave (1st degree relative)	◯	◯
Bereavement Leave	◯	◯

Comments

26. My …. hosts meetings at times that allow me to participate (i.e. during routine business hours for your division).

Select ONE response for each option provided.

	Not Applicable	Never or Rarely	Infrequently	Sometimes	Frequently	Usually or Always
Hospital Division	○	○	○	○	○	○
Hospital DOM	○	○	○	○	○	○
University Division	○	○	○	○	○	○
University DOM	○	○	○	○	○	○
Research Institute	○	○	○	○	○	○

Comments

27. How frequently do you use the following patient care strategies in your daily practice?

Select ONE response for each option provided.

	Not Applicable	Never or Rarely	Infrequently	Sometimes	Frequently	Usually or Always
E-consults through MOHLTC [1]	○	○	○	○	○	○
E-consults directly with colleagues	○	○	○	○	○	○
Email correspondence with patients	○	○	○	○	○	○
Telemedicine assessments of patients	○	○	○	○	○	○

[1] MOHLTC = Ministry of Health and Long-Term Care

Medicine
UNIVERSITY OF TORONTO

Faculty Survey 2019

Department of Medicine (DoM) Faculty Survey 2019

SECTION 4: COMMUNICATIONS

The DOM wants to reach its members in a way that is convenient and relevant. Please take a moment to answer these questions that will tell us how you want to hear from us and about the content of the communications.

28. How often do you visit the DoM website?
Select ONE response only.

- ◯ Never or Rarely
- ◯ Infrequently
- ◯ Sometimes
- ◯ Frequently
- ◯ Usually or Always

29. In which form(s) do you prefer to receive information about the DoM?
Select ALL responses that that apply.

- ☐ DoM website
- ☐ DoM Matters (monthly e-newsletter)
- ☐ Weekly Digest (email)
- ☐ Division e-newsletter
- ☐ Twitter (@UofT DoM and/or @UofT DoM Chair)
- ☐ Other (please specify)

____

30. Please indicate your level of interest in each of the following topics:

Select ONE response for each option provided.

	Definitely not Interested	Slightly Interested	Moderately Interested	Very Interested	Extremely Interested
Updates on DoM policies, procedures, strategic priorities	◯	◯	◯	◯	◯
Faculty achievements	◯	◯	◯	◯	◯
Trainee achievements	◯	◯	◯	◯	◯
Alumni achievements	◯	◯	◯	◯	◯
Upcoming deadlines (Funding opportunities, application deadlines, nomination deadlines, etc.)	◯	◯	◯	◯	◯
High-impact emerging research from our DOM	◯	◯	◯	◯	◯
Upcoming events in all divisions, specialties and/or sub-specialties	◯	◯	◯	◯	◯

Other (please specify)

[ ]

Medicine
UNIVERSITY OF TORONTO

Faculty Survey 2019

Department of Medicine (DoM) Faculty Survey 2019

SECTION 5: QUALITY IMPROVEMENT

The DoM wants to understand the impact of Quality Improvement on ALL faculty members, not just those in the Clinician in Quality and Innovation (C-QI) academic position description.

31. In the past 12 months, have you participated in any of the following?
Select ALL responses that apply.

- ☐ Led or collaborated on a QI [2] project or initiative primarily driven by academic interests
- ☐ Led or collaborated on a QI [2] project or initiative at your clinical site primarily driven by operational priorities
- ☐ Led or participated in a patient safety event investigation (e.g., root cause analysis)
- ☐ Attended an educational activity (e.g., workshop, certificate course, rounds) with an explicit focus on quality/safety?
- ☐ Participated in QI [2] as part of creative professional activity for my academic position description
- ☐ None of the above

[2] QI = Quality Improvement

32. Greater participation in QI activities by our faculty (not just by those in the CQI [3] job description) has raised questions about the academic merit of QI. Which of the following statements best captures your perspectives about QI.
Select ONE response only.

- ○ I find it hard to regard QI as having academic merit, even though I agree that QI involves fixing “broken” processes which may be valuable to hospital leaders/leadership.
- ○ I think that QI has academic merit which can be measured by related grants and publications.
- ○ I think that QI has academic merit because it represents a type of Creative Professional Activities (CPA). CPA has well-established metrics that represent external impact beyond grants and publications.
- ○ I am unsure about my views on the academic merit of QI.
- ○ None of the above capture my views. Please comment below:

[3] CQI = Clinician in Quality and Innovation

Medicine
UNIVERSITY OF TORONTO

Faculty Survey 2019

Department of Medicine (DoM) Faculty Survey 2019

SECTION 5: DEMOGRAPHICS

The DoM is collecting demographic information because of our commitment to diversity. We are tracking diversity in order to hold ourselves accountable and ensure we are reflecting the patient populations that we serve. Please note that the information you provide is anonymous and your privacy and confidentiality will be respected. The data you provide will not be linked with any other DoM databases and will only be analyzed in aggregate. Data will only be analyzed if there are >= 5 respondents in a subgroup to protect the anonymity of respondents. You can answer none, some, or all of the demographic questions.

33. Primary Hospital or Research Institute.
Select ONE response only (the site you identify with most).

- ◯ Baycrest
- ◯ Bridgepoint – Sinai Health System
- ◯ Mount Sinai Hospital – Sinai Health System
- ◯ Princess Margaret Cancer Centre – University Health Network
- ◯ Providence Healthcare – Unity Health Toronto
- ◯ Research institute appointment only
- ◯ St. Joseph's Hospital – Unity Health Toronto
- ◯ St. Michael's Hospital – Unity Health Toronto
- ◯ Sunnybrook Health Sciences Centre
- ◯ Toronto General Hospital - University Health Network
- ◯ Toronto Western Hospital - University Health Network
- ◯ Women's College Hospital
- ◯ Prefer not to answer

34. Division or Research Institute. Check your "primary" division.
Select ONE response only.

- ◯ Cardiology
- ◯ Critical Care
- ◯ Dermatology
- ◯ Emergency Medicine
- ◯ Endocrinology
- ◯ Gastroenterology
- ◯ General Internal Medicine
- ◯ Geriatrics
- ◯ Hematology
- ◯ Infectious Diseases
- ◯ Medical Oncology
- ◯ Nephrology
- ◯ Neurology
- ◯ Physical Medicine and Rehabilitation
- ◯ Respirology
- ◯ Rheumatology
- ◯ One of Allergy and Immunology, Clinical Pharmacology, Pain, Occupational Medicine, Palliative Care
- ◯ Other
- ◯ Research institute appointment only
- ◯ Not Applicable
- ◯ Prefer not to answer

35. Academic Position Description

Select ONE response only.

- ◯ Clinician Administrator (C-A)
- ◯ Clinician Educator (C-E)
- ◯ Clinician Investigator (C-I)
- ◯ Clinician in Quality and Innovation (C-QI)
- ◯ Clinician Scientist (C-S)
- ◯ Clinician Teacher (C-T)
- ◯ Research Scientist
- ◯ Prefer not to answer
- ◯ Other (please specify)

36. Rank

Select ONE response only.

- ◯ Lecturer
- ◯ Assistant Professor
- ◯ Associate Professor
- ◯ Full Professor
- ◯ Emeritus (e.g. professor, dean, president)
- ◯ Prefer not to answer
- ◯ Other (please specify)

37. Age (years).

Select ONE response only.

- ◯ <= 30
- ◯ 31 – 40
- ◯ 41 – 50
- ◯ 51 – 60
- ◯ 61 – 70
- ◯ 71 – 80
- ◯ > 80
- ◯ Prefer not to answer

38. What is your present gender identity?
Select ONE response only.

- ◯ Woman
- ◯ Man
- ◯ Trans Woman
- ◯ Trans Man
- ◯ Gender Non-Conforming
- ◯ Gender Fluid
- ◯ Two-spirit
- ◯ Prefer not to answer
- ◯ Other (please specify):

39. What is your sexual orientation?
Select ONE response only.

- ◯ Asexual/Non-sexual
- ◯ Bisexual
- ◯ Gay
- ◯ Heterosexual ('straight')
- ◯ Lesbian
- ◯ Two-spirited
- ◯ Pan-Sexual
- ◯ Not sure/Questioning
- ◯ Prefer not to answer
- ◯ Other (please specify):

40. Do you care for any dependents (this may include a child, parent, other family member, relative with a disability, etc.)?
Select ONE response only.

- ◯ Yes
- ◯ No
- ◯ Prefer not to answer

41. Do you consider yourself to be a person of colour or member of a visible minority in Canada?
Select ONE response only.

- ◯ Yes
- ◯ No
- ◯ Prefer not to answer

42. Which of the following best describes your race/ethnic background?
Select ALL responses that apply.

- ☐ Indigenous (eg. First Nations, Inuit, Métis person) – from Canada
- ☐ Indigenous – from another country
- ☐ Black – African (eg. Ghanaian, Kenyan, Somalian, etc)
- ☐ Black – North American (eg. Canadian, American) or Caribbean (eg. Barbadian, Jamaican, etc)
- ☐ Latin / Hispanic (eg. Argentinian, Chilean, Salvadorian, etc)
- ☐ East Asian (eg. Chinese, Japanese, Korean, etc.)
- ☐ South Asian (eg. Indian, Pakistani, Sri Lankan, East Indian from Guyana, etc.)
- ☐ Southeast Asian (eg. Filipino, Cambodian, Indonesian, Laotian, Vietnamese, Thai, etc.)
- ☐ West Asian (eg. Iranian, Iraqi, Persian, etc.)
- ☐ Central Asian (eg. Kazakh, Afghan, Tajik, Uzbek, Caucasus, etc.)
- ☐ Middle Eastern
- ☐ White – European (eg. British, Italian, Portugese, or Russian) or North American (eg. Canadian, American)
- ☐ Mixed heritage
- ☐ Prefer not to answer
- ☐ Other (please specify)

43. Until the age of 16, which of the following best describes your family's socio-economic status in the country you lived?
Select ONE response only.

- ○ Lower
- ○ Lower-middle
- ○ Middle
- ○ Upper-middle
- ○ Upper
- ○ Prefer not to answer

44. Whether or not it affects your day-to-day life, are you a person with adisability?
Please check ONE only.

- ○ Yes
- ○ No
- ○ Not sure
- ○ Prefer not to answer

Medicine
UNIVERSITY OF TORONTO

Faculty Survey 2019

Department of Medicine (DoM) Faculty Survey 2019

SECTION 5B: DEMOGRAPHICS \ DISABILITY

45. Is your disability ...
Select ALL responses that apply.

- ◯ Visible
- ◯ Non-visible
- ◯ Both
- ◯ Prefer not to answer

46. What type of disability do you have?
Select ALL responses that apply.

- ☐ Physical, functional and/or mobility disability (e.g., arthritis, paraplegia, cerebral palsy, muscular dystrophy, spinal cord injuries, spina bifida)
- ☐ Blind and/or low vision
- ☐ Deaf, deafened and/or hard of hearing
- ☐ Speech disability (e.g., stuttering)
- ☐ Chronic medical condition (e.g., diabetes, chronic pain, HIV/AIDS, systemic exertion intolerance disease, kidney disease, seizure disorders)
- ☐ Developmental disability (e.g., Asperger's Syndrome, Autism, Fetal Alcohol Spectrum Disorders)
- ☐ Learning disability (e.g., dyslexia)
- ☐ Psychiatric disability and/or mental health disability (e.g., bipolar disorder, obsessive compulsive disorder)
- ☐ Prefer not to answer
- ☐ Other (please specify)

____

Medicine
UNIVERSITY OF TORONTO

Faculty Survey 2019

Department of Medicine (DoM) Faculty Survey 2019

FINAL COMMENTS

47. The DoM will be engaging in strategic planning this fall to lay out priorities for the next five years. Please take a moment to tell us what you think our DoM should be emphasizing as part of our next strategic plan:

48. If you could make one change in policy or practice that would improve your workplace as a place for all to work, what would it be?

49. Are there any missing items that should be included in future iterations of this survey?

50. Are there any other comments or points on which you wish to elaborate?

Medicine
UNIVERSITY OF TORONTO

Faculty Survey 2019

Department of Medicine (DoM) Faculty Survey 2019

CONCLUSION

Over the course of the survey, you may have identified some issues of concern. Here is a list of potential resources that we hope you will utilize if you need additional supports or guidance:

a. Clinical Faculty Advocate (CFA) within the Faculty of Medicine:
https://www.deptmedicine.utoronto.ca/clinical-faculty-advocate
b. Mentorship Facilitator: https://www.deptmedicine.utoronto.ca/mentorship-facilitators
c. OMA Wellness resources including Toll-free line: http://php.oma.org/wellnessResourcesYou.html
d. DoM Leadership, University of Toronto: https://www.deptmedicine.utoronto.ca/our-leadership
