# Supplementary material for: The impact of organizational culture on professional fulfillment and burnout in an academic department of medicine
Source: PLoS One. 2021 Jun 9;16(6):e0252778. doi: 10.1371/journal.pone.0252778 (PMC8189486; doi:10.1371/journal.pone.0252778)
Supplement: Attachment — Submitted filename: Response to reviewer comments R1.docx [file pone.0252778.s002.docx]

**ONE-D-20-32481**

**The impact of organizational culture on professional fulfillment and burnout in an academic Department of Medicine**

**Submitted by Dr. Karen E. A. Burns**

**Date: Feb 23, 2021**

____________________________________________________________________

1. Please ensure that your manuscript meets PLOS ONE's style requirements, including those for file naming. The PLOS ONE style templates can be found at

https://journals.plos.org/plosone/s/file?id=wjVg/PLOSOne_formatting_sample_main_body.pdf and

<https://journals.plos.org/plosone/s/file?id=ba62/PLOSOne_formatting_sample_title_authors_affiliations.pdf>

 Done. Thank you.

2. Please include additional information regarding the survey or questionnaire used in the study and ensure that you have provided sufficient details that others could replicate the analyses.For instance, if you developed a questionnaire as part of this study and it is not under a copyright more restrictive than CC-BY, please include a copy, in both the original language and English, as Supporting Information.

 We have appended the questionnaire as Appendix 1. Thank you for this suggestion.

3. We note that you have indicated that data from this study are available upon request. PLOS only allows data to be available upon request if there are legal or ethical restrictions on sharing data publicly. For information on unacceptable data access restrictions, please see http://journals.plos.org/plosone/s/data-availability#loc-unacceptable-data-access-restrictions.

In your revised cover letter, please address the following prompts:

a) If there are ethical or legal restrictions on sharing a de-identified data set, please explain them in detail (e.g., data contain potentially identifying or sensitive patient information) and who has imposed them (e.g., an ethics committee). Please also provide contact information for a data access committee, ethics committee, or other institutional body to which data requests may be sent.

Although, we have no difficulty sharing our database, we (Department of Medicine, under the leadership (Chair) of Dr. Gillian Hawker) did not advise our full time faculty respondents that we would make their de-identified responses, including their demographic data and responses to open ended questions, accessible through an open access policy.

We do not have the opportunity to revisit this issue at this point in time as the questionnaire was administered in 2019. Since we told respondents that all data would be confidential, we feel that it would be unethical to provide the study database at this time. However, for individuals who are interested, we would be willing to consider providing access to the data on an individual basis with a written request stating the purpose and how the data would be used.

Individuals interesting in accessing the database may contact Mr. Ed Lorens, 6 Queen’s Park Crescent West, 3^rd^ Floor, Toronto, Ontario, Canada M5S 3H2; E-mail: ed.lorens@utoronto.ca

b) If there are no restrictions, please upload the minimal anonymized data set necessary to replicate your study findings as either Supporting Information files or to a stable, public repository and provide us with the relevant URLs, DOIs, or accession numbers. Please see http://www.bmj.com/content/340/bmj.c181.long for guidelines on how to de-identify and prepare clinical data for publication. For a list of acceptable repositories, please see <http://journals.plos.org/plosone/s/data-availability#loc-recommended-repositories>.

We will update your Data Availability statement on your behalf to reflect the information you provide.

 Not applicable. Please see above.

4. Thank you for stating the following financial disclosure:

'The funders had no role in study design, data collection and analysis, decision to publish, or preparation of the manuscript.'

At this time, please address the following queries:

a. Please clarify the sources of funding (financial or material support) for your study. List the grants or organizations that supported your study, including funding received from your institution.
b. State what role the funders took in the study. If the funders had no role in your study, please state: “The funders had no role in study design, data collection and analysis, decision to publish, or preparation of the manuscript.”
c. If any authors received a salary from any of your funders, please state which authors and which funders.
d. If you did not receive any funding for this study, please state: “The authors received no specific funding for this work.”

Please include your amended statements within your cover letter; we will change the online submission form on your behalf.

 Included in our cover letter. Thank you.

This study was unfunded. The authors received no specific funding for this work.

5. Thank you for stating the following in the Competing Interests section:

'Gillian Hawker receives salary support as the Sir John and Lady Eaton Professor and Chair of Medicine, Department of Medicine, University of Toronto. Sharon Straus holds a Tier 1 Canada Research Chair from the Canadian Institutes of Health. Karen Burns holds a Physician Services Incorporated Mid-Career Clinical Research Award. No other competing interests are declared.'

a. Please confirm that this does not alter your adherence to all PLOS ONE policies on sharing data and materials, by including the following statement: "This does not alter our adherence to  PLOS ONE policies on sharing data and materials.” (as detailed online in our guide for authors [http://journals.plos.org/plosone/s/competing-interests](about:blank)).  If there are restrictions on sharing of data and/or materials, please state these. Please note that we cannot proceed with consideration of your article until this information has been declared.

We confirm that this does not alter our adherence to PLOS ONE policies on data sharing and materials. We have added the following,

Gillian Hawker receives salary support as the Sir John and Lady Eaton Professor and Chair of Medicine, Department of Medicine, University of Toronto. Sharon Straus holds a Tier 1 Canada Research Chair from the Canadian Institutes of Health. Karen Burns holds a Physician Services Incorporated Mid-Career Clinical Research Award. No other competing interests are declared. We confirm that salary support and personnel awards did not impact our ability to adhere to PLOS ONE policies on data sharing and materials.

b. Please include your updated Competing Interests statement in your cover letter; we will change the online submission form on your behalf.

Done.

Please know it is PLOS ONE policy for corresponding authors to declare, on behalf of all authors, all potential competing interests for the purposes of transparency. PLOS defines a competing interest as anything that interferes with, or could reasonably be perceived as interfering with, the full and objective presentation, peer review, editorial decision-making, or publication of research or non-research articles submitted to one of the journals. Competing interests can be financial or non-financial, professional, or personal. Competing interests can arise in relationship to an organization or another person. Please follow this link to our website for more details on competing interests: [http://journals.plos.org/plosone/s/competing-interests](about:blank)

 The above text regarding potential COIs has been added to the manuscript.

6. PLOS requires an ORCID iD for the corresponding author in Editorial Manager on papers submitted after December 6th, 2016. Please ensure that you have an ORCID iD and that it is validated in Editorial Manager. To do this, go to ‘Update my Information’ (in the upper left-hand corner of the main menu), and click on the Fetch/Validate link next to the ORCID field. This will take you to the ORCID site and allow you to create a new iD or authenticate a pre-existing iD in Editorial Manager. Please see the following video for instructions on linking an ORCID iD to your Editorial Manager account: <https://www.youtube.com/watch?v=_xcclfuvtxQ>.

I have entered my ORCID ID previously. Verified.

[Note: HTML markup is below. Please do not edit.]

Reviewers' comments:

Reviewer's Responses to Questions

**Comments to the Author**

1. Is the manuscript technically sound, and do the data support the conclusions?

The manuscript must describe a technically sound piece of scientific research with data that supports the conclusions. Experiments must have been conducted rigorously, with appropriate controls, replication, and sample sizes. The conclusions must be drawn appropriately based on the data presented.

Reviewer #1: Yes

Reviewer #2: Yes

2. Has the statistical analysis been performed appropriately and rigorously?

Reviewer #1: Yes

Reviewer #2: Yes

3. Have the authors made all data underlying the findings in their manuscript fully available?

The [PLOS Data policy](http://www.plosone.org/static/policies.action#sharing) requires authors to make all data underlying the findings described in their manuscript fully available without restriction, with rare exception (please refer to the Data Availability Statement in the manuscript PDF file). The data should be provided as part of the manuscript or its supporting information, or deposited to a public repository. For example, in addition to summary statistics, the data points behind means, medians and variance measures should be available. If there are restrictions on publicly sharing data—e.g. participant privacy or use of data from a third party—those must be specified.

Reviewer #1: Yes

Reviewer #2: No

Although, we have no difficulty sharing our database, we did not advise respondents that we would make their de-identified responses, including their demographic data, accessible through an open access policy. We do not have the opportunity to revisit this issue at this point in time. Since we told respondents that all data would be confidential, we feel that it would be unethical to provide the database at this time. However, for individuals who are interested, I would be willing to consider providing access to the data on an individual written request basis.

Please see our response below

4. Is the manuscript presented in an intelligible fashion and written in standard English?

PLOS ONE does not copyedit accepted manuscripts, so the language in submitted articles must be clear, correct, and unambiguous. Any typographical or grammatical errors should be corrected at revision, so please note any specific errors here.

Reviewer #1: Yes

Reviewer #2: Yes

5. Review Comments to the Author

Please use the space provided to explain your answers to the questions above. You may also include additional comments for the author, including concerns about dual publication, research ethics, or publication ethics. (Please upload your review as an attachment if it exceeds 20,000 characters)

Reviewer #1: It was a pleasure reviewing the manuscript “The impact of organizational culture on professional fulfilment and burnout in an academic Department of Medicine”

The topic is relevant and the idea innovative. Burnout is frequent in the ICU setting and it is largely undiagnosed. The mortality rate of adult ICU patients depends on the severity of illness and patient population; it ranges between 6.4% and 80% during the COVID-19 Pandemic. Interdisciplinary work between many medical professions is essential in an ICU to deliver high quality patient care. Members of the ICU team include physicians, nurses, technicians, therapists, nutritionists, pharmacists, and other support staff making effective management an important element of a highly functioning ICU. Currently, health care delivery has far lower levels of reliability than that achieved in other industries (e.g., aviation) and staff are not supported efficiently.

Successful improvement of Critical Care requires a perspective that treats the ICU as a complex, socio-technical system and prevents staff to suffer burnout. ICU healthcare workers who provide aggressive care to critical patients have moral distress and are at risk for burnout, which in turn can lead to poor quality patient care and higher job turnover rates (Meltzer et al. Am J Crit Care 2004, Corley et al. J Adv Nurs 2000, Appropricus ESICM Abstract).In another survey 70% of ICU workers perceived conflicts, (Azoulay E, Timsit JF, Sprung CL, et al. Prevalence and Factors of Intensive Care Unit Conflicts: The Conflicus Study. Am J Respir Crit Care Med. 2009 Jul 30m). These were usually considered harmful and were significantly associated with job strain. Workload, communication, and end-of-life care emerged as potential targets for improvement.

Hamric and colleagues evaluated moral distress in both nurses and doctors. They found that ICU nurses experienced moral distress more often than physicians; however, similar situations provoked moral distress in both groups (Hamric et al. Crit Care Med 2007). Studies related to moral distress and futile or inappropriate care in the ICU do not provide patient-linked data. Consequently, the real extent of the problem of perceived inappropriate care at the end-of-life in the ICU is unknown and the magnitude of situations causing feelings of moral distress may be underestimated. Feelings like frustration, stress, guiltiness, lack of motivation, lack of communication, isolation and finally burn out are common. There is evidence in the medical and non-medical literature suggesting that the burn out leads to low performance and concentration. The performance of all Critical Care workers is prone to error and is often associated with monitoring and daily care tasks like ordering of medication or execution of patient treatment. ERRORS cause distress and can lead to more burnout. A blaming culture can lead to more mistakes and so on. The organisational culture is as complex to measure as is the incidence or severity of burnout. There are few validated tools and these are limited.

This study is looking at a relevant element and it is using one those few tools that can provide reliable information. The Stanford Professional Fulfilment Index (PFI) reports on professional fulfilment, work exhaustion, interpersonal disengagement.


Major concerns:
The limit of most surveys is the response rate which in this occasion is just above 50%.
The target people for this survey are the faculty members, in reality the entire staff could be included. The ICU is a complex system and isolating one part of the staff can have benefits and disadvantages such as reporting only on one component or one side of the problem.
The percentage of people happy with their condition was high. Results presented in this manuscript would be more valuable if a benchmark was available. This model could be used in several institutions and compared in future.

Thanks for your kind review.

Although higher response rates are achieved in single discipline surveys, a response rate of 50% is considered acceptable for multidisciplinary cross-sectional surveys. Mean response rates of 54% [1] to 61% [2] for physicians and 68%32 for nonphysicians have been reported in recent systematic reviews of postal questionnaires.

**References**

Asch DA, Jedrzwieski MK, Christakis NA. Response rates to mail surveys published

in medical journals. *J Clin Epidemiol* 1997;50:1129-36.

Cummings SM, Savitz LA, Konrad TR. Reported response rates to mailed physician

questionnaires. *Health Serv Res* 2001;35:1347-55.

We agree that all member of individual hospitals or ICUs could be surveyed with our questionnaire. This may represent an area of inquiry for future research.

Our study presents the findings of a biennial survey administered to **physicians** that are full time faculty members within our DOM. The DOM at the University is the largest DOM in Canada with over 600 full time members including physicians from a wide range of specialties. Most surveys have focused on burnout in multidisciplinary members (nurses, physicians, allied health care providers). Conversely our goal was to examine career satisfaction, professional fulfillment, burnout and the impact of organizational culture on these metrics among **physicians** from various specialties within our large academic DOM. As such this represents the largest survey ever conducted using the Stanford PFI to measure both professional fulfillment and burnout. This tool was specifically designed to assess these wellness measures in physicians. Selection of this tool, aligned well with our objective which was to examine career satisfaction, professional fulfillment, burnout and the impact of organizational culture on these metrics in physicians.

Although our findings many not be generalizable to other disciplines, we believe that our findings are generalizable to academic physicians and aligns with our intended goals.

Minor concerns:
-small error in the text and the title page : “The impact of organizational culture on professional fulfillment and burnout in an academic…”
Please change fulfilment

Fulfillment is spelled with two letter l’s throughout.

We checked the Merriam-webster dictionary to verify that this is the correct spelling.

https://www.merriam-webster.com/dictionary/fulfillment?src=search-dict-box

**Definition of *fulfillment***

**1:** the act or process of [fulfilling](https://www.merriam-webster.com/dictionary/fulfill)the *fulfillment* of a promisethe *fulfillment* of all the requirements

**2:** the act or process of delivering a product (such as a publication) to a customerthe *fulfillment* of a book order

Thank you for taking the time to review our manuscript and for your thoughtful input and review.

______________________________________________________________________

Reviewer #2: Thank you for the opportunity to review this paper by Dr. Burns and colleagues on the impact of organizational culture on professional fulfillment and burnout in an academic department of medicine. This well-written paper reports the results of a cross-sectional survey of 400 full-time physicians. I have a few comments/queries for the authors to consider:

Abstract:
1) Please provide the score range for the SPFI so the mean scores presented in the results are interpretable

Thank you for this suggestion. The score range has now been added to the abstract.

We used multivariable linear regression to examine the relationship of measures of workplace culture on professional fulfillment and burnout (scores 0-10), controlling for physician factors.

2) Are the "physician factors" referred to on line 40 the same as the "physician characteristic" defined on line 34? if so perhaps change one of the terms for consistency.

These terms represent different facets.

Physician characteristics included age (≤50, 51-60, 61-70, >70 years); gender (female/male); self-reported disability status (yes/no); under-represented minority (URM) membership (yes/no); socioeconomic status (SES) as a child/adolescent (lower/lower-middle/middle/upper-middle/upper); academic rank (lecturer/assistant professor/associate professor/professor), clinical specialty and academic position to determine protected time for scholarly work.

Physician factors include the above physician characteristics and whether respondents were interested in reducing their clinical workload to aid work-life integration (yes/no/not sure).

We have changed physician characteristics to physician factors where appropriate throughout the manuscript. Thank you for noting this.

Introduction
4) The introduction is concise and lays out both the problem and gap well.

Thanks kindly for this comment.

Methods
5) For the assessments, more detail on some of the definitions would be helpful. For example, for self-reported disability status, how was this question asked? Does it include physical, mental, or cognitive disabilities? For participant age, why were those grouping selected? 60% of respondents are in the <50 are group.

Thank you for this suggestion. We have appended the questionnaire as an electronic appendix. (see Appendix 1)

We asked whether individuals perceived that they had a disability.

**Whether or not it affects your day-to-day life, are you a person with a disability?**

***Please check ONE only.***

***Response options included Yes, No, Not sure, and Prefer not to answer***

***We defined disability in the hyperlink as follows:***

**Disability / Disabilities.**  A person with a disability is someone who has a long-term or recurring physical, mental, sensory, psychiatric or learning disability and considers oneself to be disadvantaged by reason of that disability, or believes that society is likely to consider them to be disadvantaged by reason of that disability. A person with a disability may also be someone whose functional limitations owing to their disability have been accommodated in their environment.  Examples of disabilities include, but are not limited to:

· Addiction to alcohol or drugs
· Chronic illness (e.g. epilepsy, cystic fibrosis, cancer, diabetes)
· Developmental disability (e.g. autism, down syndrome, brain injury) 
· Learning disability (e.g. dyslexia, attention deficit hyperactivity disorder (ADHD))
· Mental illness (e.g. schizophrenia, depression)
· Physical disability (e.g. cerebral palsy, spinal cord injury, amputation)
· Sensory disability (i.e. hearing or vision loss)

Regarding age, we included 4 age categories age (≤50, 51-60, 61-70, >70 years). WE collapsed the lowest three age categories due to small numbers (no one less than 30 years of age, very few between ages 31 and 40, more individuals between 41 and 50 into one category < 50 years. A similar phenomenon occurred at the higher end with only a few physicians >80 years of age. Consequently, we collapsed these individuals with physicians who were > 70 years of age. Looking at he distribution of the data, we felt that the best way to collapse the data was using the aforementioned cut offs as most respondents were between 51 and 70. Thank you for the opportunity to clarify.

Results
6) The sentence starting on line 138 is confusing and it is difficult to tell which numbers apply to which groups given the placement of comas.

Thank you for the opportunity to clarify and remove a comma.

Respondents were similar to non-respondents with respect to age group and hospital affiliation, but were significantly less likely to be a man (60.6% vs. 51.0%), assistant professor (43.6% vs. 38.2%) or cardiologist (14.4% vs. 9.2%).

The revised text reads as follows:

Respondents were similar to non-respondents with respect to age group and hospital affiliation but were significantly less likely to be a man (60.6% vs. 51.0%), assistant professor (43.6% vs. 38.2%) or cardiologist (14.4% vs. 9.2%), respectively.

Discussion
7) The paragraph (starting line 251) discussing the novel findings related to disability should be tempered slightly. With only 12 people in this group the results should be interpreted with caution.

We have changed the wording to acknowledge that this finding should be regarded as hypothesis generating. Thank you for this suggestion.

First sentence: We found a relationship between having a disability and both lower professional fulfillment and higher burnout scores.

Current sentence:

Although limited to 12 respondents, we found a relationship between having a disability and both lower professional fulfillment and higher burnout scores.

We believe that the remaining text tempers our findings and is aligned with the reviewer’s comments. Thank you.

To our knowledge no prior study has reported these relationships. Unlike gender and URM status, the relationships between disability and both professional fulfillment and burnout were not attenuated by factors related to organizational culture suggesting that the effect of disability on both metrics may be through different mechanisms related to environmental barriers [35] or the effort required by disabled physicians to complete work-related tasks.

We have also edited the last sentence in this paragraph.

Additional research is needed to confirm these findings in disabled physicians and if true to ascertain how more inclusive work environments can be created for disabled physicians.

Thank you Dr. Rubulotta and Reviewer #2. We sincerely appreciate you taking the time to review our manuscript.

While revising your submission, please upload your figure files to the Preflight Analysis and Conversion Engine (PACE) digital diagnostic tool, <https://pacev2.apexcovantage.com/>. PACE helps ensure that figures meet PLOS requirements. To use PACE, you must first register as a user. Registration is free. Then, login and navigate to the UPLOAD tab, where you will find detailed instructions on how to use the tool. If you encounter any issues or have any questions when using PACE, please email PLOS at [figures@plos.org](mailto:figures@plos.org). Please note that Supporting Information files do not need this step.

Not applicable.
